# Characterization of the Complete Mitogenome of the Ring-Necked Pheasant *Phasianus colchicus* (Galliformes: Phasianidae) and Systematic Implications for Phasianinae Phylogenetics

**DOI:** 10.3390/genes15121569

**Published:** 2024-12-04

**Authors:** Qinggang Mei, Yiming Deng, Dongmei Zhao, Daoyu Jiang, Yaqing Liao, Xiangmei Yu, Peng Liu, Lichun Jiang

**Affiliations:** 1 Key Laboratory for Molecular Biology and Biopharmaceutics, School of Life Science and Technology, Mianyang Teachers’ College, Mianyang 621000, China; scmeiqg@163.com (Q.M.); shach37519@163.com (Y.D.); 17808309011@163.com (D.Z.); jdy07173395@163.com (D.J.); 17873081786@163.com (Y.L.); 15520567392@163.com (X.Y.); 2 Ecological Security and Protection Key Laboratory of Sichuan Province, Mianyang Teachers’ College, Mianyang 621000, China

**Keywords:** *Phasianus colchicus*, mitochondrial genome, phylogenetic analysis, divergence time, Ka/Ks

## Abstract

Background: Phasianidae mitogenomes exhibit significant structural variations critical for understanding evolution and subspecies divergence. However, annotations of these features in some pheasant species remain limited. This study aimed to enhance understanding of Phasianidae mitogenomes and their evolutionary patterns. Methods: A comparative analysis of complete mitogenomes from *Phasianus colchicus*, *Phasianus versicolor*, and 22 other accipitrids was conducted, examining codon usage, rRNA structures, selective pressures, phylogenetics, and structural variations. Results: The mitogenome of *P. colchicus* is 16,696 bp, comprising 13 protein-coding genes, 2 rRNA genes, 22 tRNA genes, and a control region, with a base composition of A: 30.61%, T: 25.26%, C: 30.85%, and G: 13.28%. Phylogenetic analysis revealed *P. colchicus* and *P. versicolor* are closely related, with the following relationship: ((*Phasianus* + *Chrysolophus*) + (*Crossoptilon* + *Lophura*)). Divergence timing aligns with the Tibetan Plateau uplift during the Tertiary Pliocene. Ka/Ks analysis suggests the *CO I*, *CO II*, *CO III*, *ND1*, *ND4L*, and *ND6* genes in *Phasianus* underwent strong selective pressure for plateau adaptation. Conclusions: The study confirms *Phasianus monophyly* and its close relationship with Chrysolophus. Adaptation-related selective pressures on the *CO I*, *CO II*, *CO III*, *ND1*, *ND4L*, and *ND6* genes highlight its role in plateau environments, offering valuable insights into pheasant phylogeny.

## 1. Introduction

The ring-necked pheasant, scientifically known as *Phasianus colchicus*, is a species of bird that is widely recognized and valued for its significance in both research and wildlife management [1,2]. This species, which is part of the Galliformes order and the Phasianidae family, is a prevalent game bird with a distribution that spans across the globe. Its ubiquity and status as a common game bird underscore its importance on an international scale. The common pheasant, a globally distributed resident species, originates from the temperate zones of the Palearctic region, stretching from the Russian Far East to the eastern and southeastern parts of Europe, beyond the Black Sea, and extending south to Indochina and Afghanistan [3,4]. This bird holds significant economic value, with a storied history of being released for hunting purposes and maintained in captivity within bird farms across Western Europe, North America, and Australia [3]. Its widespread presence and economic importance have made the common pheasant a key species in both recreational hunting and aviculture. The common pheasant’s widespread distribution and adaptability render it an ideal subject for exploring critical issues in speciation, the evolution of traits, biogeography, and local adaptation to diverse climates. Additionally, it serves as a valuable model for studies in wildlife management and conservation genetics, providing insights into the complex interplay between species evolution and environmental adaptation.

The mitochondrion, a prevalent organelle in the majority of eukaryotic cells, plays a pivotal role in energy production, accounting for over 95% of the energy supply through its involvement in the oxidative phosphorylation process of the respiratory chain. This organelle possesses its own genetic material and operates an independent genetic system, classifying it as semi-autonomous. The mitochondrial genome’s attributes—small size, maternal inheritance, rapid evolutionary rate, and a scarcity of introns—make it an invaluable tool in various fields of study. It is extensively utilized in phylogenetic evolution research among related species, tracing maternal lineages, examining the connections between geographical distribution and species origins, and understanding natural hybridization and introgression events [3,4].

In recent years, significant advancements have been made in the study of avian mitochondrial genomes, with nearly 2000 bird species having their mtDNA data catalogued in the NCBI database. This information is crucial for understanding species evolution, population genetics, and taxonomy. The *Phasianus* genus, a part of the Phasianidae family within the Galliformes order, encompasses two distinct species: *P. colchicus* and *P. versicolor*. While these species share a similar body morphology, they exhibit a significant variation in feather coloration, marking a notable divergence between them. Previous studies of the common pheasant have mainly focused on its ecology and biology. The classification and systematic categorization of the common pheasant have been subjects of intense scholarly interest and ongoing research for an extended period. However, there remains a limited body of research concerning the sequence characteristics and systematic taxonomic status of *P. colchicus*. Previous research primarily focused on the simple sequence characteristics of the mitochondrial genome of *P. colchicus* and did not delve into the systematic taxonomic relationships within the Phasianidae family [5,6]. Furthermore, discussions regarding the relationship between *P. colchicus* and closely related species in Phasianidae were also absent in these articles. The taxonomic status and phylogenetic position of Phasianus are controversial. Shen et al.’s research findings suggested that the systematic classification status of these four genera is as follows: (([*Chrysolophus*, *Phasianus*], *Lophura*), *Syrmaticus*) [7], while other researchers believe that *Crossoptilon*, *Lophura*, and *Phasianus* are closely related to the evolution of *Chrysolophus* ((*Chrysolophus*, *Phasianus*), (*Lophura*, *Crossoptilon*)) [4,8]. To encapsulate, the genera *Crossoptilon*, *Lophura*, *Chrysolophus*, and *Phasianus* display a spectrum of morphological and distributional diversity. However, existing studies, which have typically focused on individual genes or gene fragments, fall short of providing a thorough and nuanced comparative analysis. This limitation underscores the need for more holistic and integrated research approaches.

Of late, mitochondrial genomes have increasingly been employed to resolve complex phylogenetic relationships [9,10,11,12]. In general, mtDNA accumulates mutations at a relatively faster rate than nuclear DNA, thus making it particularly useful for revealing closely spaced branching events. Considering that previous phylogenetic studies based on a single gene or a few genes failed to resolve the internal branching orders within the Phasianidae birds, we sequenced the complete mitochondrial genome of *P. colchicus*, further analyzed its structure, and then compared it with *P. versicolor*. Additionally, we obtained other mitogenomes from GenBank. Consequently, we used extensive mitogenome data from major groups of Phasianidae birds to infer their phylogenetic relationships.

## 2. Materials and Methods

### 2.1. Sample Collection and DNA Extraction

Specimens of *P. colchicus* were collected from Mojia Town, Fucheng County, Mianyang City, Sichuan Province, China (105°8′24.14″, 31°58′19.35″, at an elevation of 555 m above sea level). Tissue samples (2 mL) were extracted and preserved in heparin anticoagulant tubes prior to the experiment. No live animal experiments were conducted in this study. All samples were stored at −20 °C. Total genomic DNA was extracted from liver tissues using the standard phenol/chloroform method [13].

### 2.2. PCR Amplification and Sequencing

By PCR amplification and LA-PCR amplification, 12 overlapping fragments covering the entire mtgenome were obtained, each overlapping by 150–250 bp. The complete mtDNA was amplified into a concatenated sequence by 7 pairs of primers and selectively amplified mtDNA templates obtained from the literature [14]. The remaining five PCR primer pairs were designed using Premier 5.0 and based on the comparison of relatively conserved nucleotide sequences of six homologous Phasianidae species in GenBank. The reaction volume of PCR amplification was 25 μL. It contains 2.5 μL 10× loading buffer, 2.0 μL MgCl_2_ (2.5 mol/L), 1.5 μL dNTP mixture (2.5 mM/L each), 1.0 μL (10 μmol/L) per pair of primers, 1.0 μL DNA template (20 ng/µL), 0.6 μL LA Taq polymerase (5 U/µL), and sterilized water. The cycle steps included an initial denaturation of 2.5 min (94 °C), denaturation of 45 s (94 °C), annealing of 40 s (50–61 °C), extension of 80–180 s (72 °C), and final extension of 9 min (72 °C). All PCR products were detected using 1% agarose gel electrophoresis and then subjected to Sanger sequencing. The details of the PCR primers are listed in Table S1.

### 2.3. Mitochondrial Genome Annotations and Sequence Analysis

DNA sequences were edited and assembled manually. The locations of protein-coding genes (PCGs), tRNAs, and rRNA genes were annotated via BLAST alignment to search for homologous sequences, which were then used as reference sequences. We used the same method to compare the reference sequence with homologous DNA sequences for determining the control region using BioEdit (version: 7.0.5.2) [15]. The MITOS web server was used to annotate the mitochondrial genomes [16] followed by NCBI ORFfinder (https://www.ncbi.nlm.nih.gov/orffinder, accessed on 29 September 2024) and BLAST (https://blast.ncbi.nlm.nih.gov, accessed on 29 September 2024), which were used to validate the annotations. A mitochondrial genome map was constructed using OGDRAW (https://chlorobox.mpimp-golm.mpg.de/OGDraw.html, accessed on 1 October 2024) with default parameters [17]. The tRNA secondary structures were analyzed by tRNAscan-SE (https://trna.ucsc.edu/tRNAscan-SE/index.html, accessed on 2 October 2024) [18] and MITOS web servers (http://mitos.bioinf.uni-leipzig.de/, accessed on 2 October 2024) with the default search mode, using the vertebrate mitochondrial genetic code source. The R2DT online tool (https://r2dt.bio/, accessed on 2 October 2024) was used to predict the tRNA structure. Nucleotide composition, relative synonymous codon usage (RSCU) values, and genetic divergence between species were calculated using the software MEGA (version 11.0, https://www.megasoftware.net/, accessed on 5 September 2024) [19]. The AT and GC skewness were calculated as follows: AT skew = (A − T)/(A + T) and GC skew = (G − C)/(G + C) [20]. The overlapping regions and intergenic spacers between the genes were manually calculated.

### 2.4. Phylogenetic Analysis

Phylogenetic datasets with 12 PCGs (*ATP6*, *ATP8*, *CO I*, *CO II*, *CO III*, *ND1*, *ND2*, *ND3*, *ND4*, *ND4L*, *ND5*, and *ND6*), except for *CYTB* (b) from 64 sequences were analyzed, including 38 Phasianinae, 3 Arborophilinae, 13 Pavoninae, 8 Odontophoridae, and 2 outgroup species (*Anser cygnoides* and *Tadorna ferruginea*). The accession numbers of the sequences are listed in Table S2. The amino acid sequences of the 12 PCGs were aligned using MAFFT in PhyloSuite v.1.2.3 with default parameters [21,22]. The Gblocks program in PhyloSuite (version 1.2.3) was used to clear the ambiguously aligned fragments of the 12 PCGs sequences. Phylogenetic trees were constructed based on the alignment dataset using maximum likelihood (ML) and Bayesian inference (BI) methods conducted by MEGA (version 11.0) and the MrBayes (version 3.2.6) plugin integrated into PhyloSuite (version 1.2.3), respectively [19,23]. The ML tree was inferred using the parameters of 1000 bootstrap replications. The best-fitting model of the BI phylogenies was estimated by ModelFinder (http://www.iqtree.org/ModelFinder/, accessed on 5 October 2024) with the params of 5,000,000 generations [24]. The plot of the phylogenies tree was drawn with the tool FigTree (http://tree.bio.ed.ac.uk/software/figtree/, accessed on 5 October 2024).

### 2.5. Divergence Time Estimates Focused on Phasianus and Chrysolophus

Based on the 13 PCGs of each species, we estimated the divergence time with the BEAST (version 1.10.4, https://beast.community/, accessed on 5 October 2024) program [25]. The GTR evolutionary model and the strict molecular clock were applied in estimating the timescale. The Yule process was used as the tree prior. Fossil calibration points were set as follows: *Arborophila ardens* and *Arborophila rufipectus* split 11.6–14.4 Mya; *Gallus gallus* and *Gallus sonneratii* split 7.1–9.6 Mya; *Lagopus lagopus* and *Lagopus muta japonica* split 3.1–13.3 Mya. The MCMC chain was run for 20,000,000 generations, with the first 10% discarded as burn-in. Parameters were logged every 2000 generations, and the ESS values were checked using Tracer (version 1.7.2, http://tree.bio.ed.ac.uk/software/tracer/, accessed on 5 October 2024) [26], with all ESS values exceeding 200. The tree was generated using TreeAnnotator v1.8.2 (https://beast.community/treeannotator, accessed on 5 October 2024), and divergence times were visualized with FigTree v1.4.5 (http://tree.bio.ed.ac.uk/software/figtree/, accessed on 5 October 2024).

### 2.6. Ka and Ks Analysis

The degree of selection against non-synonymous substitutions, in contrast to synonymous ones, is quantified by the Ka/Ks ratio. This ratio is determined by comparing the frequency of non-synonymous substitutions at non-synonymous sites (Ka) with that of synonymous substitutions at synonymous sites (Ks). To discern the variations in the evolutionary trajectory of sequences between species, we computed the Ka/Ks distributions through pairwise DNA sequence comparisons for each of the 12 protein-coding genes (PCGs), utilizing a Ka/Ks calculator (version 2.0, https://ngdc.cncb.ac.cn/tools/kaks, accessed on 5 October 2024). This methodical approach facilitates a nuanced analysis of the evolutionary patterns, highlighting the distinct selective pressures that have influenced the genetic sequences across different species [27].

## 3. Results

### 3.1. Mitogenomic Structure and Comparison

**Figure 1:**
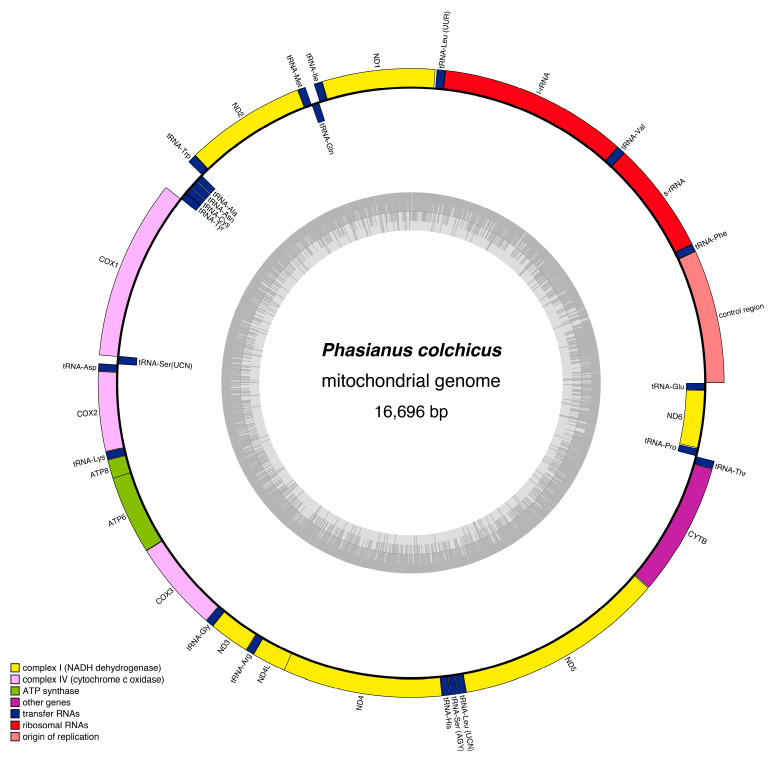
Structural map of the *P. colchicus* mitogenome. The 38 genes are encoded across both DNA strands. Protein-coding genes and those for rRNAs are represented using standard abbreviations. For tRNAs, a single letter corresponds to the specific amino acid they carry, with numerical distinctions made for the two types of leucine tRNAs and the two types of serine tRNAs. This methodical labeling ensures a clear and organized representation of the genetic components.

We have successfully deposited the complete mitogenome of *P. colchicus* in the GenBank database under the accession number PQ474154. The mitogenome sequence of *P. colchicus* spans 16,696 base pairs (bp), as detailed in Table 1 and illustrated in Figure 1. This size places *P. colchicus* within the typical range observed for fully sequenced Phasianidae species, with lengths varying from a minimum of 16,488 bp in *Caloperdix oculeus* to a maximum of 16,841 bp in *Gallus lafayetii*, as summarized in Table S2. The alignment analysis has identified 38 distinct mitogenome regions in *P. colchicus*, comprising 13 protein-coding genes (PCGs), 2 ribosomal RNAs (rRNAs), 22 transfer RNAs (tRNAs), and a non-coding region (D-loop) characterized by a high adenine (A) and thymine (T) content—a feature prevalent in the mitochondrial DNA (mtDNA) of most Phasianidae birds, as outlined in Table 1. The light strand (L-strand) of *P. colchicus*’s mitogenome is responsible for encoding one PCG and eight tRNAs, whereas the majority of the genes are located on the heavy strand (H-strand), as depicted in Table 1 and Figure 1.

**Figure 2:**
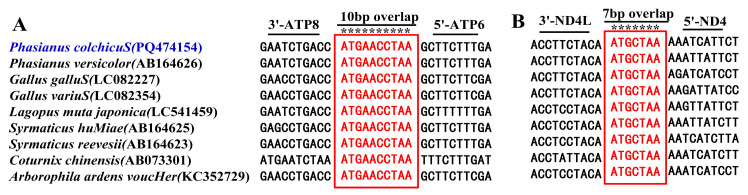
Sequence alignments reveal the conservation of motifs in the *ATP8*/*ATP6* and *ND4L*/*ND4* regions of Phasianidae avian species. (**A**): *ATP8* and *ATP6* overlap region; (**B**): *ND4L* and *ND4* overlap region. The highlighted nucleotides delineate the highly conserved 10 bp (ATGAACCTAA) and 7 bp (ATGCTAA) overlaps among these avian species. The “*” symbol represents the conservative sites.

Species within the same genus usually have the same characteristics. The overall nucleotide composition of *P. colchicus* was A (30.61%), T (25.26%), C (30.85%), and G (13.28%); while that of *P. versicolor* was A (30.70%), T (25.26%), C (30.86%), and G (13.18%). Both *Phasianus* species exhibit a positive AT skew and a negative GC skew across their entire mitogenomes. Indeed, regions abundant in A + T are considered the primary drivers of variation in mitogenome length. During polynucleotide operations, including insertion and deletion, the quantity of nucleotides fluctuates, and the number and types of tandem repeat elements significantly differ among species. This genetic diversity underscores the complexity of mitogenome evolution [28]. We identified two long gene overlaps in *P. colchicus* and *P. versicolor*, namely, a 10-base-pair (bp) overlap (ATGAACCTAA) and a 7-bp overlap (ATGCTAA), both of which are common among numerous Phasianidae species and, as depicted in Figure 2, are situated between ATP8 and ATP6 and between ND4L and ND4 on the H-strand, respectively. These overlapping sequences are posited to function as a bicistronic message, indicating that they are translated across gene boundaries [29,30,31]. After comparative mitogenomic analysis, the sequence motifs located between *ATP8* and *ATP6*, as well as between *ND4L* and *ND4*, were found to be relatively conserved among Phasianidae species. Our findings reveal that gene overlap regions are a pervasive feature in the mitochondrial genomes of eukaryotes. The presence of these gene overlap regions allows for a more efficient and economical use of genomic sequences, enabling the encoding of additional genetic information, which is crucial for the transmission of genetic data across species [31].

### 3.2. PCGs

The base composition of the 13 protein-coding genes (PCGs) in the mitogenomes of *P. colchicus* and *P. versicolor* aligns closely with that observed in other Phasianidae birds. For *P. colchicus*, the AT content of these 13 PCGs ranged from 50.51% to 59.39%, while *P. versicolor* exhibited a range of 51.85% to 58.79%. Both species demonstrated analogous biases towards A and T nucleotides, reflecting a shared genetic trait within the Phasianidae family (Table 2). Among the protein-coding genes in the mitogenomes of *P. colchicus* and *P. versicolor*, the ND4L gene displayed the lowest AT content, while the ATP8 gene had the highest. The initiation and termination codon usage in *P. colchicus* mirrored that of *P. versicolor*. The exception was the start codon of *CO I*, which was GTG, contrasting with the ATG start codon found in the other genes. Regarding termination codons, *ND2*, *ND4*, and *CO III* genes ended with an incomplete T. Notably, the *ND2* gene terminated with a TAG codon, while *ND6* and *CO I* genes ended with both TAG and AGG codons, and the rest of the genes concluded with TAA. This pattern underscores the subtle variations in codon usage within the Phasianidae species.

The codon analysis of the 13 protein-coding genes (PCGs) in *P. colchicus* and *P. versicolor* revealed a total of 3796 codons, with the absence of the AGA termination codon. Among these, the codons CUA, AUC, and UUC were the most frequently utilized, occurring 259, 204, and 167 times in *P. colchicus* and 270, 207, and 157 times in *P. versicolor*, respectively. This pattern underscores the codon usage bias in the mitogenomes of the two *Phasianus* species. In terms of the amino acid composition encoded by the 13 protein-coding genes (PCGs), *P. versicolor* uniquely encoded an additional Leu, Ile, Pro, Ala, His, Lys, Glu, Arg, Gly, and Ser compared to *P. colchicus*. Conversely, *P. colchicus* had a higher count of valine (Val), proline (Pro), threonine (Thr), lysine (Lys), arginine (Arg), and glycine (Gly) than *P. versicolor*. This comparative analysis highlights the subtle differences in the genetic encoding of amino acids between the two species (Figure S1).

**Figure 3:**
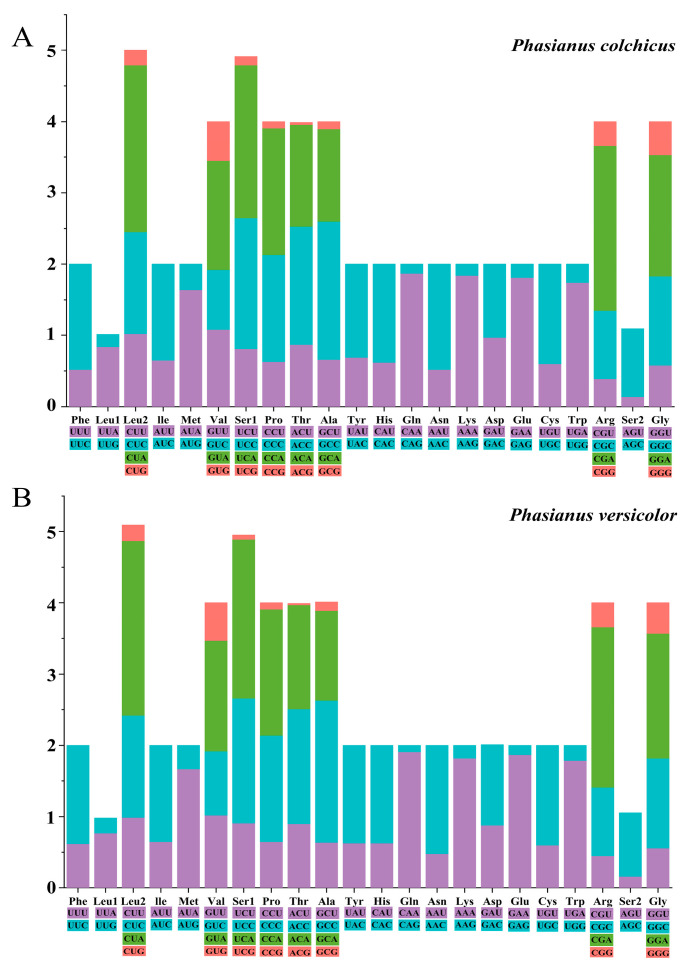
Relative Synonymous Codon Usage (RSCU) in *P. colchicus* (**A**) and *P. versicolor* (**B**) mitogenome. Codon families are provided on the X axis and the RSCU values on the Y axis.

Upon examining the RSCU values of the two species, subtle differences were observed in the frequency of synonymous codon usage for various amino acids, as depicted in Figure 3. For instance, in *P. versicolor*, the codon CUA was more prevalent in the Leu2 codon than in *P. colchicus*. Conversely, *P. colchicus* exhibited a higher frequency of the codon UUC in Phe compared to *P. versicolor*. These findings highlight the nuanced variations in codon preference between the two Phasianidae species. The analysis of amino acid variations within the ATP6 gene of *P. colchicus* and *P. versicolor* reveals distinct genetic fingerprints. Specifically, at the 111th position, *P. colchicus* presents leucine (Leu), while *P. versicolor* exhibits phenylalanine (Phe). Furthermore, at the 191st position, alanine (Ala) is observed in *P. colchicus*, contrasting with threonine (Thr) found in *P. versicolor*.

### 3.3. Transfer RNAs and Ribosomal RNAs

**Figure 4:**
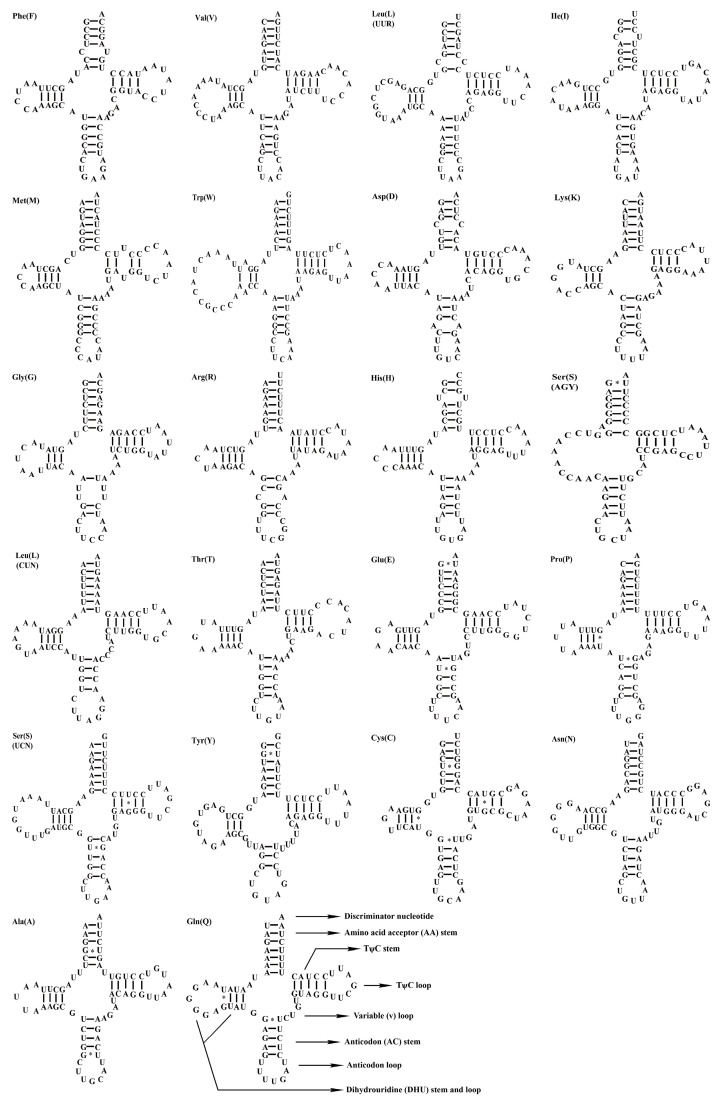
The secondary structural predictions for the 22 tRNA genes in *P. colchicus.* “*” represents a weak key.

In the mitogenome of *P. colchicus*, a total of twenty-two tRNAs were identified, with two being repetitive: tRNA-Leu and tRNA-Ser (as detailed in Table 1). Figure 4 illustrates the secondary structure of these tRNAs, which exhibit sequence lengths varying from 67 to 75 base pairs. Upon analysis of their predicted secondary structures, it was observed that tRNASer (AGN) lacks the dihydrouracil (DHU) loop, thereby preventing it from forming a complete cloverleaf configuration. Conversely, the remaining tRNAs were found to be capable of forming the standard cloverleaf secondary structure. The fundamental features of tRNA in *P. versicolor* mirror those of *P. colchicus*. Both species exhibit the conventional A-U and G-C base pairing within their mitochondrial tRNA secondary structures, as depicted in Figure 4 and Figure S1. Notably, there are 16 and 15 instances of the non-canonical G-U wobble base pairs in *P. colchicus* and *P. versicolor*, respectively. The majority of these wobble pairs are located in the amino acid acceptor stem (with five occurrences in each species) and the anticodon stem (six in *P. colchicus* and five in *P. versicolor*). Furthermore, the DHU arm harbors three G-U pairs in each species, while the TψC arm contains two pairs. Additionally, other types of mismatched bases, such as U-U, C-U, C-C, A-A, and A-C, are also present (as shown in Figure 4). Concurrently, Varani and Mcclain have posited that the G-U mismatched base pairs may significantly contribute to the stability of tRNA’s secondary structure [32].

**Figure 5:**
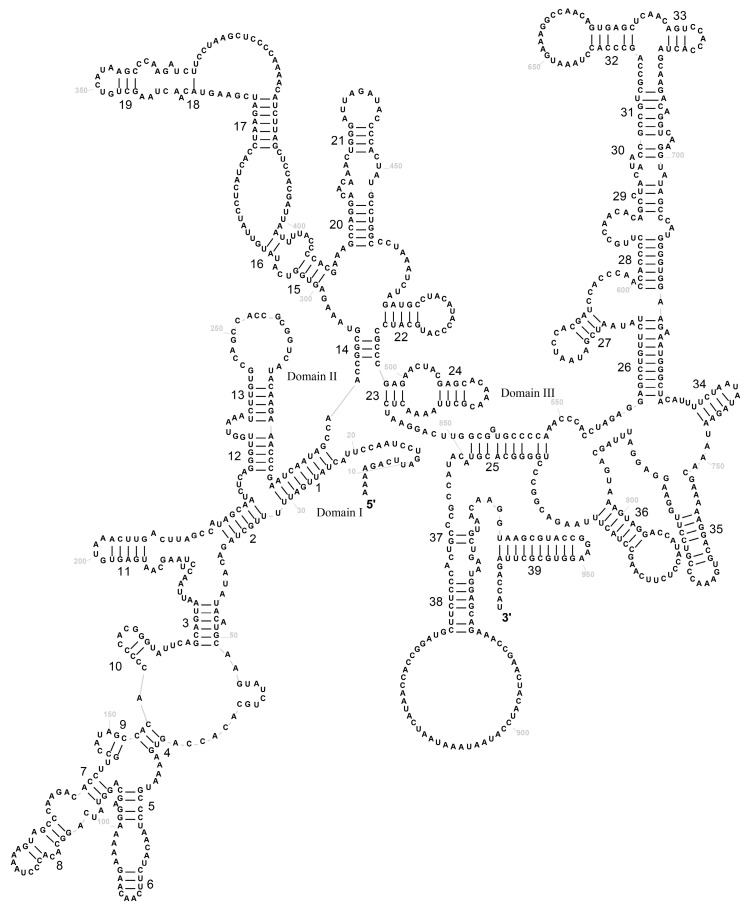
The prognostic map of 12S rRNA secondary structures in *P. colchicus*.

**Figure 6:**
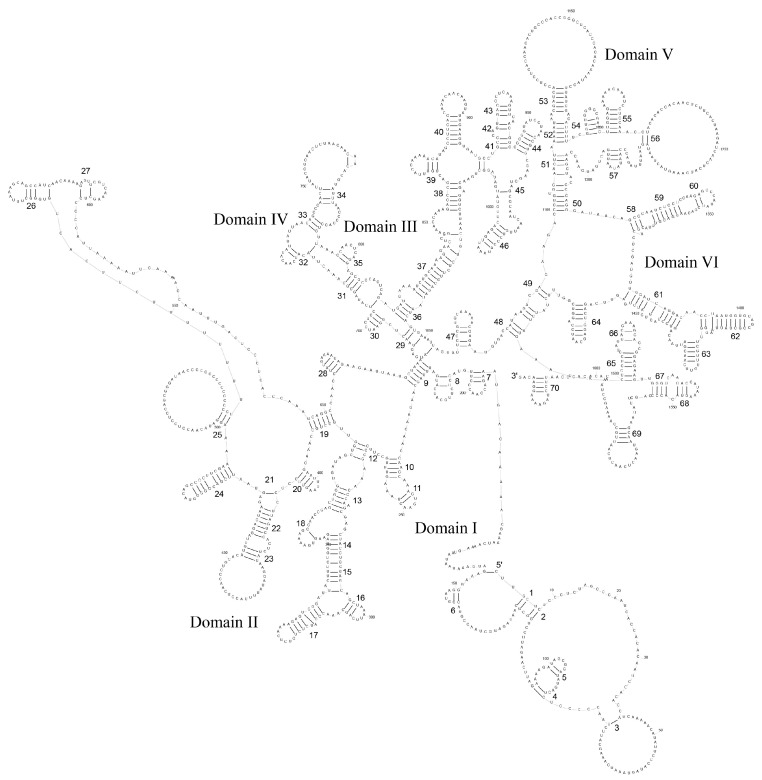
The prognostic map of 16S rRNA secondary structures in *P. colchicus*.

In the mitochondrial genomes of *P. colchicus* and *P. versicolor*, two types of rRNA are present: 12S rRNA and 16S rRNA. The 12S rRNA is situated between the tRNA-Phe and tRNA-Val genes, and both species share a 12S rRNA sequence length of 966 base pairs, with a divergence of 11 base pairs. The 16S rRNA, positioned between tRNA-Val and tRNA-Leu (UUR), exhibits a length of 1626 base pairs in *P. colchicus* and 1620 base pairs in *P. versicolor*, with a total of 26 base pair differences. The predicted secondary structures of the 12S and 16S rRNAs are composed of three domains with 39 stem–loop structures and six domains with 70 stem–loop structures, respectively (Figure 5 and Figure 6).

### 3.4. Control Region

**Figure 7:**
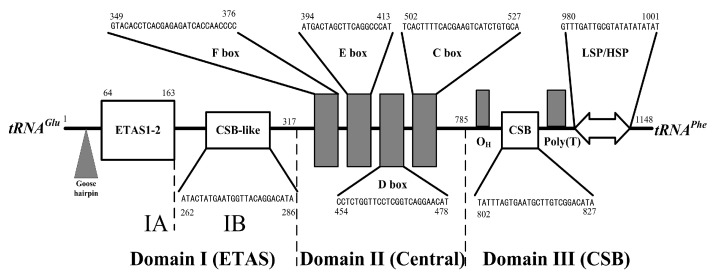
Schematic representation of the organization of the *P. colchicus* control region. ETAS denotes the extended termination-associated sequences, while the F through C boxes refer to the conserved sequence boxes that reside in the central domain. The OH marks the origin of H-strand replication, CSB stands for conserved sequence block, and CSB-like signifies a sequence that closely resembles the CSB. Additionally, LSP is designated as the light-strand transcription promoter, and HSP is the heavy-strand transcription promoter.

The mitochondrial control region, delineated by the variable nucleotide positions and their differential frequencies, is segmented into three distinct domains as illustrated in Figure 7. In the case of *P. colchicus*, the control region’s nucleotide composition is characterized by 27.44% adenine (A), 32.49% thymine (T), 25.87% cytosine (C), and 14.20% guanine (G). This composition reveals a notable underrepresentation of guanine, a trait commonly observed in the sense strand of vertebrate mitochondrial DNA [33]. Domain I, known as extended termination-associated sequences (ETAS), comprises two segments, Part A and Part B. Part A contains a highly conserved block (5′-TACCCCCCCTTTCCCCCCCAGGGGGGGTA-3′), which shares sequence homology with the “goose hairpin” as reported in *Anas caerulescens* by Quinn [34]. Within Part A, ETAS1 and ETAS2 are located at positions 64–126 and 124–163 bp, respectively, with an overlap of 3 bp and sequence similarities of 70.3% and 45.6% to the consensus mammalian ETAS1 and ETAS2 [15]. In Part B (nt 163–317), a CSB1-like block (5′-ATACTATGAATGGTTACAGGACATA-3′) is present, exhibiting a 69.2% similarity to CSB1 in domain III. The central domain II (nt 318–785) features four conserved sequence boxes labeled C, D, E, and F. Domain III (nt 786–1148) contains a poly(C) sequence (nt 778–791), analogous to the origin of H-strand replication (OH) in mammals, which is situated just downstream of the proposed CSB1 (nt 802–827). Notably, the CSB domain of *P. colchicus* lacks distinct traces of CSB2 and CSB3. Additionally, the bidirectional light- and heavy-strand transcription promoters (LSP/HSP) are identified in *P. colchicus* (Figure 7).

### 3.5. Phylogenetic Analyses

**Figure 8:**
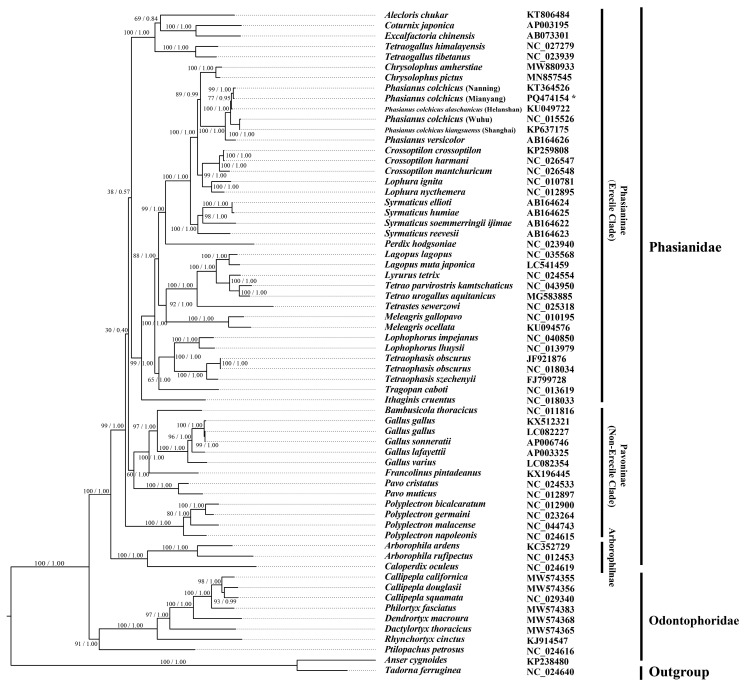
Inferred phylogenetic relationships based on PCG sequence with BI and ML methods. *A. cygnoides* (KP238480) and *T. ferruginea* (NC_024640) were used as outgroups. Bayesian posterior probability and Bootstrap value of each node are shown in turn, such as 1.00/100. The “*” represents the current study.

Mitogenome sequences of *P. colchicus* and *P. versicolor* were compared with those of 62 other species belonging to three subfamilies (Phasianinae, Rollulinae, and Pavoninae) and one family (Odontophoridae). Phylogenetic trees were meticulously constructed employing maximum likelihood (ML), Bayesian inference (BI), and neighbor-joining (NJ) methodologies, with *A. cygnoides* and *T. ferruginea* designated as the outgroup species. The phylogenetic trees derived from these three approaches exhibited minor discrepancies, yet the BI tree demonstrated greater confidence levels (as depicted in Figure 8). Excluding the outgroup comprising *A. cygnoides* and *T. ferruginea*, the remaining 62 species from Pheasinidae and Odontophoridae were categorized into four distinct branches. Based on the phylogenetic tree results, Pheasinidae is divided into three subfamilies, Phasianinae, Rollulinae, and Pavoninae. *Ptilopachus petrosus* is clustered in the Odontophoridae family, and it is recommended to place it in this family (Figure 8). In these clusters, *Lophophorus* and *Tetraophasis* were gathered into a branch, and *Crossoptilon*, *Chrysolophus*, *Lophura*, *Phasianus*, *Syrmaticus*, and *Perdix* were clustered into a branch in both of the BI and ML trees [4]. The evolutionary ties between *Crossoptilon* and *Lophura* were found to be rather intimate, aligning with the findings of prior research [35]. This study’s outcomes substantiated that *Chrysolophus* is genetically affiliated with both *Crossoptilon* and *Lophura*, a conclusion that harmonizes with certain contemporary viewpoints [36]. However, this finding contrasts with other studies that propose *Chrysolophus* and *Phasianus* should be grouped together within a single clade. And their research results support such a systematic relationship (*Phasianus,* (*Chrysolophus*, [*Lophura*, *Crossoptilon*])). The phylogenetic proximity of *Chrysolophus amherstiae* and *Chrysolophus pictus* is more intimate than that of other species, aligning with contemporary taxonomic classifications.

### 3.6. Phylogeny and Divergence Time of the Phasianus and Chrysolophus Species

**Figure 9:**
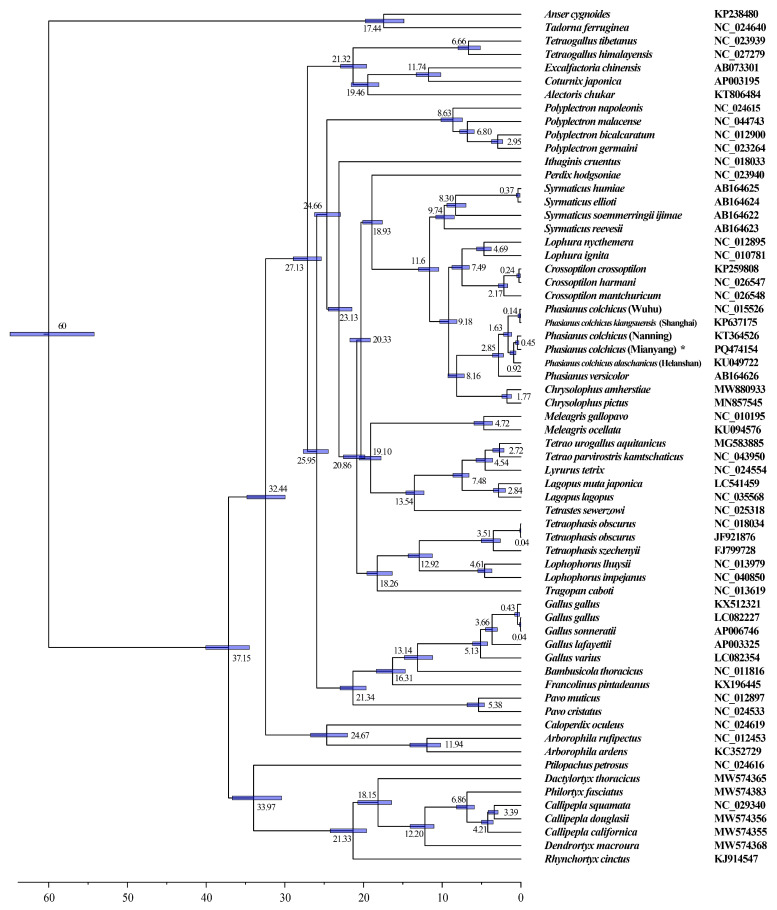
The divergence time of Phasianidae with 95% highest posterior probability density. Numbers nearby nodes refer to divergence times. The “*” represents the current study.

Within Phasianidae, the topology ((*Crossoptilon* + *Lophura*) + (*Phasianus* + *Chrysolophus*)) was supported in both the ML and BI trees, consistent with previous studies. Using a strict molecular clock, the maximum clade credibility tree suggests that the primary clade members diverged in the Early Miocene, approximately 35.34 million years ago (Mya) (Figure 9). The divergence between *Phasianus* and *Chrysolophus* occurred around 7.73 Ma, slightly earlier than the split between *Lophophorus* and *Tetraophasis* 12.56 Ma. The divergence time for *Lophura* and *Crossoptilon* was estimated at approximately 7.03 Ma. Notably, the split between *Crossoptilon crossoptilon* and *Crossoptilon harmani* occurred around 0.24 Ma, which is consistent with a previous report [37]. Additionally, *Phasianus* species collected from different geographical locations exhibited similar levels of divergence, suggesting a generally consistent origin across these populations.

### 3.7. Non-Synonymous and Synonymous Substitution

To assess the selective pressures acting on the mitochondrial protein-coding genes of *P. colchicus*, we analyzed the non-synonymous (Ka) and synonymous (Ks) substitution rates across 12 protein-coding genes. The Ka/Ks ratio values of most genes were found to be less than 1, indicating strong purifying selection acting on most genes. Notably, the ATP8 and ND3 genes exhibited slightly lower Ka/Ks ratios compared to other genes, suggesting a more conserved function in these regions. Meanwhile, the Ka/Ks values of CO I, CO II, CO III, ND1, ND4L, and ND6 genes were significantly higher than that of other comparison groups, suggesting potential functional divergence in these regions (Table S3). When compared to other Phasianinae species, the Ka/Ks patterns in *P. colchicus* were generally conserved, reinforcing its close evolutionary relationship within the group. These results contribute to understanding the evolutionary dynamics of Phasianinae mitogenomes and have systematic implications for resolving the phylogenetic relationships within this subfamily.

## 4. Discussion

### 4.1. Mitogenome Characteristics

In the two *Phasianus* mitogenomes, the highest A + T content was observed in domain III of the CR region, while the lowest one existed in the stems of tRNAs. According to the A + T content in PCGs, the second codon position revealed the highest value rather than the third codon position. The variation in AT content across different segments of the mitogenome could be attributed to two transcription-dependent processes that differentially affect mutation rates on the transcribed and non-transcribed strands of genes: transcription-coupled repair and deamination [38,39]. The third codon position in PCGs revealed A skew and C skew, therefore, PCGs were likely to include A or C as their third codon position. T skew existed in the second codon position in PCGs, indicating the preferential selection of a T base in this position. There was also T skew in domain II of the CR region, which was the most conservative region among the three domains.

Loop regions usually have a fast evolutional rate with larger differences among species, while stem regions are relative conservative [40], which may be due to different selection pressures. In a comparison of RNA secondary structures of two *Phasianus* mitogenomes, the stem regions, being essential in maintaining the secondary structure of RNA, were also more conservative than loop regions. There were also a large number of G-U mismatches in tRNAs, which also played an important role in maintaining the stability of tRNA secondary structure. Most nucleotide differences in the tRNAs of the two Phasianus mitogenomes were transitions, consistent with the findings of previous studies [36].

### 4.2. Phylogenetic Relationship and Divergence Time of the Phasianus and Chrysolophus Species

The study of phylogenetic relationships and divergence times among species within the genus *Phasianus* offers a glimpse into the evolutionary history and diversification patterns of these birds. In our research, phylogenetic analyses utilizing diverse datasets consistently endorse the monophyletic status of *Phasianus* and underscore the intimate genetic kinship between *P. colchicus* and *P. versicolor* [37]. From the perspective of the phylogenetic tree, the juvenile family is divided into three subfamilies, Phasianinae (erectile clade), Rollulinae, and Pavoninae (non-erectile clade), which is consistent with previous research results [41,42,43,44,45,46]. From the constructed phylogenetic tree, it can be seen that *P. petrosus* was clustered in a large branch with the Odontophoridae species, while Jetz et al.’s [47] research suggests that this species belonged to the Pheasinidae family. The topology presented by Jetz et al. (2012) [48] exhibited the lowest resolution, a limitation likely attributable to the misclassification of *P. nahani*. Specifically, Jetz et al. (2012) [48] erroneously assigned this species to an incorrect family, a mistake that was later rectified by Wang et al. (2017) [48]. Our topology suggests placing the *Ptilopachus* species in the Odontophoridae family, which is consistent with the previous research findings [42,43].

Our analysis confirms that the variation between different geographical samples of *Phasianus* species is relatively small. Consequently, four *Phasianus* variants can be well grouped into one clade, further indicating their close relationship. Additionally, the phylogenetic analysis of *Phasianus* species typically reveals a well-resolved evolutionary tree, exhibiting clear cladistic structures that reflect shared ancestry and genetic relatedness.

In our study, we found the split times among *P. colchicus* (Mianyang), *P. colchicus* kiangsuensis (Shanghai), *P. colchicus* (Nanning), *P. colchicus* alaschanicus (Ningxia), and *P. colchicus* (Wuhu) were relatively close. Their systematic evolutionary relationship is (([*P. colchicus* Mianyang, *P. colchicus* Nanning], *P. colchicus* alaschanicus Ningxia), (*P. colchicus* kiangsuensis Shanghai, *P. colchicus* Wuhu)), and their systematic evolutionary relationship is consistent with their geographical location. Additional samples are needed for in-depth research and analysis on their subspecies differentiation and species formation [41].

In addition, the divergence time between *P. colchicus* and *P. versicolor* occurred 2.88 Mya, which is consistent with previous reports [37,49]. These results highlighted the value of mitochondrial genome-based phylogenies, especially in situations where generating large autosomal genomic datasets is not feasible. The Miocene epoch, characterized by its warmer global climate and the emergence of kelp forests and grasslands, likely offered optimal conditions for the expansion and diversification of *P. quails* [50]. Our study’s findings are in harmony with previous research that estimated the divergence of the Phasianidae family during the mid-to-late Miocene, which is also supported by the significant climate changes and the development of new biomes that occurred during this period. These environmental shifts are believed to have played a crucial role in the diversification and range expansion of Phasianidae, including *P. quails*. Furthermore, the mid-Miocene expansion of temperate broadleaf deciduous forests is thought to have played a significant role in promoting the diversification and the subsequent geographical distribution enlargement of Phasianidae.

### 4.3. Selective Pressure on Protein-Coding Genes in Phasianus and Chrysolophus Species

In the realm of evolutionary biology, understanding the selective pressures acting on PCGs within species is crucial for elucidating their adaptive strategies and evolutionary trajectories. The *Phasianus* species, a diverse group of birds, provide an intriguing context for studying such pressures. Purifying selection, which acts to remove deleterious mutations from the genome, is often the dominant force shaping the evolution of PCGs. In *Phasianus*, evidence suggests that the majority of PCGs are under strong purifying selection, as indicated by Ka/Ks ratios consistently less than one (*p*-value < 0.001). This indicates that the amino acid sequences of these genes are highly conserved, reflecting their critical roles in maintaining essential biological functions and fitness, which is similar to previous research findings in *Crossoptilon* [37].

However, it is also evident that not all PCGs in *Phasianus* are equally constrained by purifying selection. Some genes, such as those involved in metabolic pathways or immune responses, may experience positive selection. Positive selection can lead to rapid evolution and the emergence of new traits or adaptations. In the case of *Phasianus*, specific PCGs like *CO I*, *CO II*, *CO III*, *ND1*, *ND4L*, and *ND6* genes have been shown to exhibit Ka/Ks ratios suggesting strong positive selection in certain comparison groups (KT364526 vs. PQ474154, and NC_015526 vs. KU049722) (Table S3). These genes, often related to energy metabolism and oxidative phosphorylation, may be undergoing adaptive evolution in response to environmental changes or pressures from pathogens. The observed variations in selective pressures across PCGs in *Phasianus* could be attributed to several factors. These include differences in functional constraints, where some genes are more critical for survival and reproduction than others, and the varying intensity of environmental pressures across different habitats and ecological niches. Furthermore, the evolutionary history of *Phasianus*, including its speciation events and gene flow patterns, may also influence the distribution of selective pressures across its genome.

In conclusion, the study of selective pressures on PCGs in *Phasianus* species reveals a complex interplay between purifying and positive selections, shaping the evolutionary landscape of these birds. Understanding these pressures not only enhances our knowledge of *Phasianus* biology but also provides insights into the general mechanisms of evolutionary adaptation in species. Future research should continue to explore the genetic and environmental factors driving these selective pressures, as well as their implications for the conservation and management of *Phasianus* populations.

## 5. Conclusions

To encapsulate, the mitogenomes of the *Phasianus* species exhibit congruent gene arrangements and analogous compositions, encompassing base content and secondary structural features, which is reflective of their phylogenetic affinity. Based on the dendrograms, *P. colchicus* and *P. versicolor* form sister groups. *Phasianus* have a closer relationship with *Chrysolophus*. In the context of phylogenetic relationships, *P. colchicus* and *P. versicolor* exhibit a closer affinity than other species, which aligns with current taxonomic classifications. Molecular dating estimates suggest that the divergence time between the two species coincides with the uplift of the Tibetan Plateau and the subsequent climatic shifts during the Tertiary Pliocene. Furthermore, the Ka/Ks analysis indicates that several genes, including *CO I*, *CO II*, *CO III*, *ND1*, *ND4L*, and *ND6*, in *Phasianus* species may have been under strong selective pressure to adapt to the plateau environment.

## Figures and Tables

**Table 1:** Annotation of the mitochondrial genome of *P. colchicus* (P. c) and *P. versicolor* (P. v).

Gene	Strand	Nucleotide Number		Size (bp)		IGS (bp)		Codon	
		P. c	P. v	P. c	P. v	P. c	P. v	Start	Stop *
D-loop	H	1–1148	1–1050	1148	1050	0	0		
tRNA-Phe	H	1149–1216	1151–1217	68	67	−1	0		
12S rRNA	H	1216–2181	1218–2183	966	966	0	0		
tRNA-Val	H	2182–2256	2184–2257	75	74	0	0		
16S rRNA	H	2257–3882	2258–3877	1626	1620	−1	−1		
tRNA-Leu	H	3882–3955	3877–3950	74	74	11	11		
ND1	H	3967–4941	3962–4936	975	975	0	0	ATG	TAA
tRNA-Ile	H	4942–5013	4937–5008	72	72	6	6		
tRNA-Gln	L	5020–5090	5015–5085	71	71	−1	−1		
tRNA-Met	H	5090–5158	5085–5153	69	69	0	0		
ND2	H	5159–6197	5154–6194	1039	1041	0	−2	ATG	T--/TAG
tRNA-Trp	H	6198–6275	6193–6270	78	78	2	2		
tRNA-Ala	L	6278–6346	6273–6341	69	69	3	2		
tRNA-Asn	L	6350–6422	6344–6417	73	74	2	2		
tRNA-Cys	L	6425–6491	6420–6486	67	67	0	−1		
tRNA-Tyr	L	6492–6561	6486–6556	70	71	1	1		
COI	H	6563–8113	6558–8108	1551	1551	−9	−9	GTG	AGG
tRNA-Ser	L	8105–8179	8100–8174	75	75	2	2		
tRNA-Asp	H	8182–8250	8177–8244	69	68	1	2		
COII	H	8252–8935	8247–8930	684	684	1	1	ATG	TAA
tRNA-Lys	H	8937–9004	8932–8999	68	68	1	1		
ATP8	H	9006–9170	9001–9165	165	165	−10	−10	ATG	TAA
ATP6	H	9161–9844	9156–9839	684	684	−1	−1	ATG	TAA
COIII	H	9844–10,627	9839–10,622	784	784	0	1	ATG	T--
tRNA-Gly	H	10,628–10,696	10,624–10,691	69	68	0	0		
ND3	H	10,697–11,048	10,692–11,043	352	352	1	1	ATG	TAA
tRNA-Arg	H	11,050–11,118	11,045–11,113	69	69	0	0		
ND4L	H	11,119–11,415	11,114–11,410	297	297	−7	−7	ATG	TAA
ND4	H	11,409–12,786	11,404–12,781	1378	1378	0	0	ATG	T--
tRNA-His	H	12,787–12,855	12,782–12,850	69	69	0	1		
tRNA-Ser	H	12,856–12,922	12,852–12,917	69	66	0	0		
tRNA-Leu	H	12,923–12,993	12,918–12,987	71	70	0	0		
ND5	H	12,994–14,811	12,988–14,805	1818	1818	4	4	ATG	TAA
CYTB	H	14,816–15,958	14,810–15,952	1143	1143	1	1	ATG	TAG
tRNA-Thr	H	15,960–16,028	15,954–16,023	69	70	2	1		
tRNA-Pro	L	16,031–16,099	16,025–16,094	69	70	6	5		
ND6	L	16,106–16,627	16,100–16,621	522	522	1	1	ATG	TAG
tRNA-Glu	L	16,629–16,696	16,623–16,690	68	68	0	0		

* represents stop codon. The notation “T--” signifies an incomplete termination codon. “IGS” denotes the intergenic space, which is characterized by a positive value for gaps in nucleotides or the presence of a negative value for overlapping nucleotides between adjacent genes. The designations “H” and “L” denote genes that are transcribed from the heavy and light mitochondrial strands, respectively. This nomenclature provides a clear and systematic way to describe the genetic structure and transcription orientation.

**Table 2:** Nucleotide composition of different partitions from two *Phasianus* (P. c and P. v) mitogenomes.

Genes	T (%)		C (%)		A (%)		G (%)		A + T (%)		G + C (%)		AT Skew		GC Skew	
	P. c	P. v	P. c	P. v	P. c	P. v	P. c	P. v	P. c	P. v	P. c	P. v	P. c	P. v	P. c	P. v
Mitogenome	25.26	25.26	30.85	30.86	30.61	30.7	13.28	13.18	55.47	55.96	44.53	44.04	0.0958	0.0971	−0.3981	−0.4014
PCGs	27.07	26.87	31.53	31.72	28.4	28.61	13	12.8	55.47	55.48	44.53	44.52	0.024	0.0314	−0.4161	−0.425
PCG—1st	23.01	20.12	30.25	32.71	31.23	32.74	15.51	14.43	54.24	52.86	45.76	47.14	0.1515	0.2387	−0.3221	−0.3878
PCG—2nd	31.42	31.23	31.01	28.92	23.84	23.68	13.73	16.17	55.26	54.91	44.74	45.09	−0.1372	−0.1375	−0.3862	−0.2828
PCG—3rd	26.77	29.26	33.32	33.52	30.15	29.42	9.76	7.8	56.92	58.68	43.08	41.32	0.0594	0.0027	−0.5469	−0.6225
tRNA	28.03	28.23	20.74	20.57	30.75	30.95	20.48	20.25	58.78	59.18	41.22	40.82	0.0463	0.046	−0.0063	−0.0078
rRNA	20.9	21.04	27.24	27.14	33.81	33.72	18.05	18.1	54.71	54.76	45.29	45.24	0.236	0.2316	−0.2029	−0.1998
12S rRNA	19.67	20.19	28.47	28.05	32.92	32.92	18.94	18.84	52.59	53.11	47.41	46.89	0.2519	0.2397	−0.201	−0.1964
16S rRNA	21.64	21.54	26.51	26.6	34.34	34.2	17.51	17.66	55.98	55.74	44.02	44.26	0.2269	0.2271	−0.2045	−0.202
CR	32.49	32.43	25.87	25.91	27.44	27.13	14.2	14.52	59.93	59.57	40.07	40.43	−0.0843	−0.0891	−0.2913	−0.2817
CR—Domain I	29.97	29.15	27.44	27.9	30.28	30.41	12.3	12.54	60.25	59.56	39.75	40.44	0.0052	0.0211	−0.381	−0.3798
CR—Domain II	31.62	32.05	31.41	30.98	16.67	16.67	20.3	20.3	48.29	48.72	51.71	51.28	−0.3097	−0.3158	−0.2149	−0.2083
CR—Domain III	35.81	35.81	17.36	17.63	38.84	37.74	7.99	8.82	74.66	73.55	25.34	26.45	0.0406	0.0262	−0.3696	−0.3333

P. c: *P. colchicus*; P. v: *P. versicolor*.

## Data Availability

Mitochondrial genome sequence data supporting the findings of this study are openly available from GenBank of the National Center for Biotechnology Information (NCBI) at https://www.ncbi.nlm.nih.gov (accessed on 20 October 2024) (accession number: PQ474154).

## Supplementary Materials

The following supporting information can be downloaded at: https://www.mdpi.com/article/10.3390/genes15121569/s1, Figure S1: Predicted secondary structures for the 22 tRNA genes of *P. versicolor*; Table S1: PCR primers covering the mitochondrial genome of *P. colchicus*; Table S2: Mitogenomes of the Phasianidae used in the study; Table S3: The Ka/Ks values among the *Phasianus* species.
