# Supplementary figures and images for: Characterization of the Complete Mitogenome of the Ring-Necked Pheasant Phasianus colchicus (Galliformes: Phasianidae) and Systematic Implications for Phasianinae Phylogenetics
Source: Genes (Basel). 2024 Dec 4;15(12):1569. doi: 10.3390/genes15121569 (PMC11675856; doi:10.3390/genes15121569)

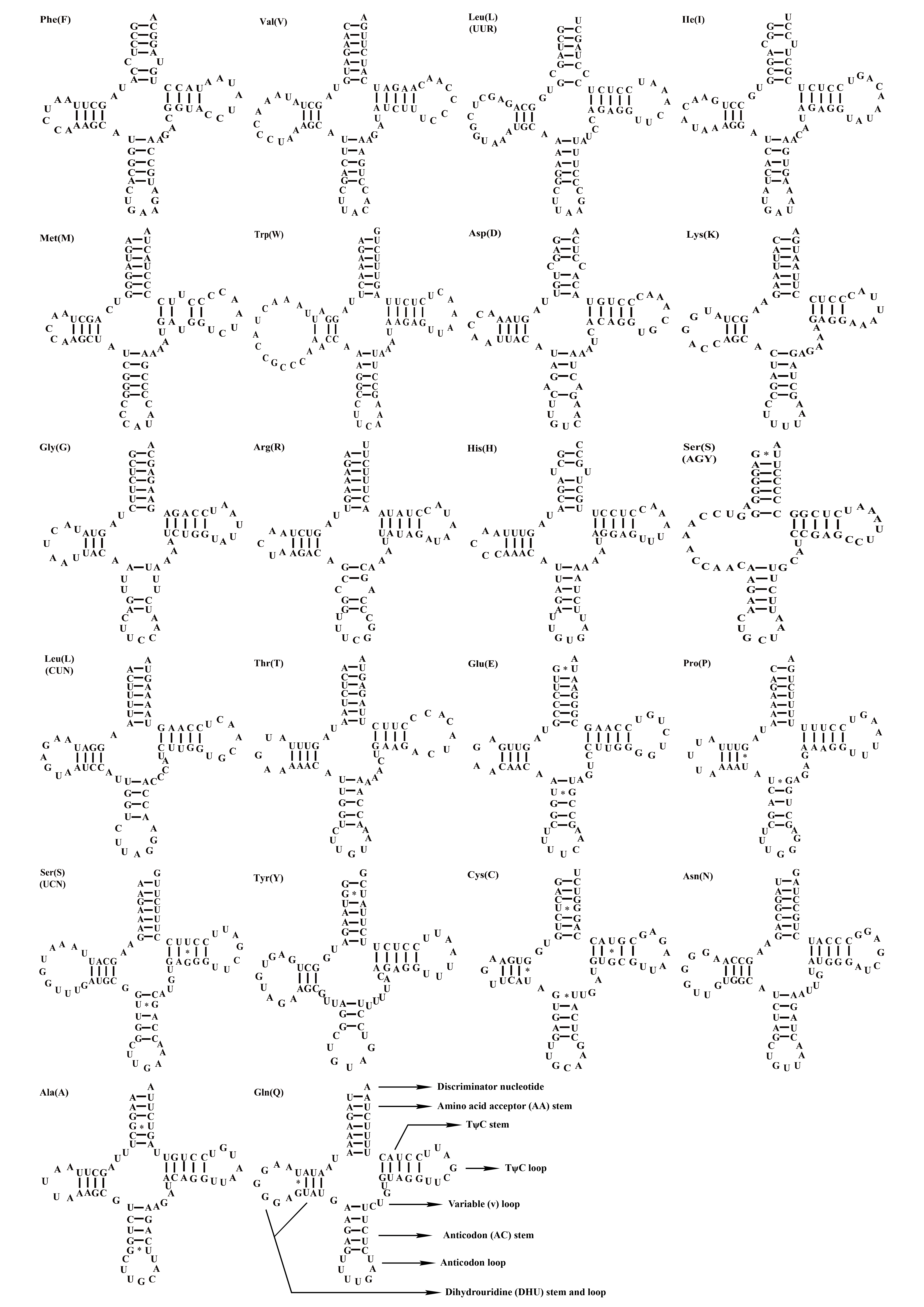

Supplement: Supplementary file 1 [file genes-15-01569-s001.zip › Figure._S1.jpg]
